# Influence of Prefecture-Level Yield of Not-for-Sale Vegetables on Vegetable Intake in Japan: A Natural Experiment

**DOI:** 10.3390/nu14142884

**Published:** 2022-07-14

**Authors:** Daisuke Machida

**Affiliations:** 1 Home Economics Education Course, Cooperative Faculty of Education, Gunma University, 4-2, Aramaki, Maebashi 371-8510, Gunma, Japan; machi@gunma-u.ac.jp; Tel.: +81-27-220-7344; 2 Faculty of Agriculture, Takasaki University of Health and Welfare, 37-1, Nakaorui, Takasaki 370-0033, Gunma, Japan

**Keywords:** difference-in-differences, food environment, health promotion, natural experiment, nonmarket food, not-for-sale vegetables, vegetable intake

## Abstract

Increased vegetable intake contributes to better health for people. The distribution of not-for-sale vegetables is an important source of vegetable intake in Japan. This study examined the impact of prefecture-level yield of not-for-sale vegetables on vegetable intake in Japan. This study regarded the increase in yield of not-for-sale Chinese cabbage in Nagano Prefecture in 2012 as a natural experiment. The years 2012 and 2016 were the large-scale survey years of the Japanese National Health and Nutrition Survey. Therefore, the effect of the change in prefecture-level yield of not-for-sale vegetables on vegetable intake was evaluated by comparing the changes in Chinese cabbage intake in Nagano between 2012 and 2016 with those of other prefectures classified in the same regional block as Nagano. Statistical analysis was performed using general linear models to examine the interaction of year and prefecture with Chinese cabbage intake. Consequently, the regression coefficient for the interaction term was −3.38 (95% CI, −9.59–2.83), that of the model adjusted for basic characteristics and energy intake was −2.99 (95% CI, −9.22–3.24), and that of the model adding health-related variables was −5.03 (95% CI, −12.40–2.34). The prefecture-level yield of not-for-sale vegetables typically had a minor effect on vegetable intake.

## 1. Introduction

Numerous studies have shown that increased fruit and vegetable intake is effective in preventing certain chronic diseases and reducing the risk of death from them [1,2,3,4,5,6,7]. For example, it has been suggested that the consumption of the functional components found in Blueberry and Alliums is effective in the prevention of many chronic diseases [1,2]. In addition, evidence from epidemiological studies indicates that the overall intake of fruits and vegetables contributes to the prevention of certain chronic diseases, namely, cardiovascular diseases, cancer, and mental illness, and a reduction in mortality [3,4,5,6,7]. The World Health Organization recommends that people consume 400 g of fruits and vegetables per day to promote good health [8].

Fruit and vegetable gardening is often used as a strategy to increase people’s fruit and vegetable intake. Numerous studies have analyzed the relationship between the use of school, community, and home gardens and fruit and vegetable intake [9,10,11,12,13,14,15,16,17]. Positive associations between gardening activity and fruit and vegetable intake were reported in many review studies [9,10,11,12,13,14]. Some reviews found no increase in fruit and vegetable intake or had inconsistent results; however, these were studies with children or with school gardens as the intervention setting [15,16,17]. Thus, although studies with children or school gardens have inconsistent results, there is generally a positive association between gardening and fruit and vegetable intake, especially among adults.

In rural Japan, in addition to self-production such as gardening, free giving and receiving of food is a common practice. Consequently, the impact of nonmarket foods on food intake cannot be ignored. On Hachijo Island, it was estimated that nonmarket foods account for approximately 25% of the production price basis, and approximately 17% of the caloric basis within food consumption [18]. Furthermore, it was assumed that approximately half of all food consumed was from nonmarket sources in seasons from the spring to autumn [19]. Hachijo Island is a particularly striking example, as it is a small island in the Pacific with a small population. However, other regions of Japan have also reported that nonmarket food is associated with diet, especially vegetable intake. A study conducted in rural Hokkaido found that the intake of vegetables received from neighbors was positively related with total vegetable intake [20]. Furthermore, in multiple locations in Gunma Prefecture, positive relationships have been confirmed among vegetable cultivation, vegetable reception and vegetable intake [21,22].

Therefore, I hypothesize that residents of areas with a high yield of not-for-sale fruits and vegetables consume a higher amount of fruits and vegetables. The harvested not-for-sale fruits and vegetables are first consumed by the grower’s household. Surplus vegetables and fruits are subsequently offered to neighbors. Neighbors who receive an excess of vegetables may offer their surplus to other neighbors. In this type of ecosystem, residents of neighborhoods with high yields of not-for-sale fruits and vegetables are likely to consume more fruits and vegetables. Previous studies have highlighted that the higher the yield of not-for-sale fruits and vegetables, the higher the intake of fruits and vegetables by residents [23,24]. However, these studies are ecological and cross-sectional, and further research is required to clarify causal relationships.

This report aims to predict the effect of changes in yields of not-for-sale vegetables by prefecture on vegetable intake in Japan. The vegetable yields used in this study were those recorded in national statistics in Japan. These figures do not include vegetables produced in home and community gardens. However, a previous study in Japan found that 96% of farmers grow vegetables for their own consumption and 84% distribute the vegetables they grow to their neighbors [22].

## 2. Materials and Methods

### 2.1. Data

This study used two forms of national statistical data in Japan. The first was the National Crop Survey (NCS). The NCS clarified the actual conditions of crop production and shipment and prepared materials to formulate production targets and promote various measures in the food, agriculture, and rural basic plans [25]. The NCS surveyed the crop acreage, yield, and shipment amount of all agricultural product shipping organizations in the target prefectures [25]. The second was the National Health and Nutrition Survey (NHNS). The NHNS investigated Japanese citizens’ physical condition, nutrient intake, and lifestyle. In addition, it obtained basic data to comprehensively promote people’s health, based on the Health Promotion Law [26,27]. The details of both of surveys are as described in the previous study [24].

### 2.2. Study Design

The natural experiment is an analytical method for identifying the effect of a specific factor on a treatment from an event in which several factors have complex influences by utilizing a change in circumstances that occurred by chance. It has been used in many studies in recent years [28,29,30]. In this study, I treated the changes in yield of not-for-sale Chinese cabbage and cabbage due to the supply–demand adjustment in 2012 as a natural experiment. In 2012, the largest shipment restraint in recent years in Japan was implemented for summer Chinese cabbage and summer/autumn cabbage under the supply–demand adjustment [31]. This policy was adopted because high crop yields resulted in lower market prices. This has resulted in an increase in the yield of Chinese cabbage and cabbage that are not-for-sale.

In Japan, over 80% of summer Chinese cabbages are produced in Nagano Prefecture, and over 50% of summer/autumn cabbages are produced in Gunma Prefecture [25].

In addition, Nagano and Gunma Prefecture are in same area group in Japan (*Kanto* 2) [26,27]. This area group includes three prefectures other than Nagano and Gunma. Thus, I treated Nagano and Gunma as an experimental group and other prefectures in *Kanto* 2 as the control group. In addition, Gunma was included in the control group when Nagano was used as the experimental group and Nagano was included in the control group when Gunma was used as the experimental group.

To determine whether the increased yield of not-for-sale Chinese cabbage and cabbage in Nagano and Gunma prefectures in 2012 affected the intake of Chinese cabbage and cabbage, I used NHNS data from 2012 and 2016 [26,27]. Only in 2012 and 2016 have large-scale surveys of the NHNS been conducted to date, with approximately five times as many responses as medium-scale surveys in other years. Fortunately, in 2012, the harvest of not-for-sale Chinese cabbage and cabbage increased in Nagano and Gunma prefectures. This was due to supply and demand adjustments [31]. Therefore, this study estimated the effect of change in prefecture-level yield of not-for-sale vegetables on vegetable intake by comparing the change in Chinese cabbage and cabbage intake from 2012 to 2016 in the experimental and control groups (difference-in-differences).

### 2.3. Subjects

The subjects were men and women aged 20–79 years living in the *Kanto* 2 area. Those who did not respond to food intake status and pregnant and lactating women, whose diets may have been significantly affected, were excluded from the analysis. In addition, subjects with energy intakes below 800 kcal and over 3000 kcal (approximately mean ± 2 SD) were excluded. Information on subjects is presented in Table 1.

### 2.4. Outcomes

I used data for Chinese cabbage and cabbage intake calculated by the NHNS. In Japan, Chinese cabbage is often eaten as a pickle, and in NHNS, pickled Chinese cabbage is classified as pickled leaves. This is tabulated separately from Chinese cabbage intake [26,27]. Therefore, when considering the intake of Chinese cabbage as an outcome, I used the intake of Chinese cabbage and the total intake of Chinese cabbage and pickled leaves as outcomes.

### 2.5. Analyses

#### 2.5.1. Trend of Yield of Not-for-Sale Chinese Cabbage and Cabbage

The trend of the yield of not-for-sale Chinese cabbage and cabbage was calculated from NCS data. The trends for the surrounding 10 years (2009–2018), including the 2012 and 2016 data for the analysis, were output for Chinese cabbage and cabbage. Similar to previous studies [23,24] that used NCS data, the not-for-sale yield was defined and calculated as the amount harvested minus the amount shipped by prefectures (ton/year). Furthermore, the not-for-sale yield was divided by the population of each prefecture (national census data [32]) and the number of days in a year, and ton was converted to gram (×10^6^) to calculate the value as the not-for-sale yield per person per day.

#### 2.5.2. Confirmation of Parallel Trends

When conducting difference-in-differences analysis, it is a prerequisite that the trends of the outcome variables are parallel between the experimental and control groups before the period of interest. However, since large-scale surveys of the NHNS were conducted twice, it is difficult to directly confirm whether parallel trends existed between the experimental and target groups before 2012. Therefore, this study tentatively confirmed the parallel trend by referring to a previous study by Suzuki et al. [33]. I used data from *Kanto* 1, located close to *Kanto* 2 in the regional classification of the NHNS [26,27], to confirm the parallel trend with the *Kanto* 2 control group as a convenient alternative.

General linear models were used in the analyses. Independent variables were the survey year (2012 (ref.) and 2016), area (*Kanto* 2 (ref.) and *Kanto* 1), and their interaction terms. Dependent variables were Chinese cabbage intake, total intake of Chinese cabbage and leafy pickles, and cabbage intake. The regression coefficients (95% confidence intervals (CIs)) were calculated. Four prefectures belong to *Kanto* 1. The information of the subjects in *Kanto* 1 is shown in Table A1. For the *Kanto* 2 subjects, the analyses were performed excluding Nagano or Gunma as the experimental group.

#### 2.5.3. Main Analyses (Difference-in-Differences)

I examined whether there was a difference in the change in each intake from 2012 to 2016 between the groups. The survey year, prefecture, and their interaction terms were the independent variables, and each intake was the dependent variable. Three models were used in the analyses. Model 1 was a crude model. Model 2 was a model adjusted for basic characteristics (sex, age, living style, and employment) and energy intake (quartile groups). Model 3 adjusted for health-related indicators (body mass index, alcohol drinking) in addition to Model 2. Regression coefficients (95% CIs) and estimated means (95% CIs) were calculated for each model.

#### 2.5.4. Software and Statistical Significance

All analyses were conducted using IBM SPSS Statistics for Windows, version 28.0 (IBM Japan, Ltd., Tokyo, Japan), and Python 3.9.7 was used to create the figures.

The statistical significance level was not set due to instructions from the Ministry of Health, Labour and Welfare when applying for data use because the sample size was too small for proper prefecture-level comparisons.

### 2.6. Ethical Approval

The NHNS data were obtained with permission from the Ministry of Health, Labour and Welfare. The NCS data were obtained from the Japanese official statistics portal site e-stat, and these data did not contain any personally identifiable information. This study was approved by Takasaki University of Health and Welfare Research Ethics Committee (No.: 1907; Approval date: 31 May 2019).

## 3. Results

### 3.1. Trends of Yield of Not-for-Sale Chinese Cabbage and Cabbage

**Figure 1:**
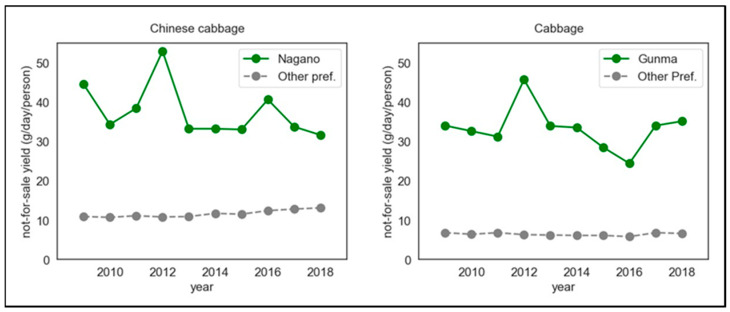
Trends of yield of not-for-sale Chinese cabbage and cabbage. Nagano: only Nagano Prefecture; Gunma: only Gunma Prefecture; Other pref.: for Chinese cabbage, mean of prefecture among *Kanto* 2 other than Nagano (i.e., Ibaraki, Tochigi, Gunma, and Yamanashi), and for cabbage, mean of prefecture among *Kanto* 2 other than Gunma (i.e., Ibaraki, Tochigi, Yamanashi, and Nagano). Details of the data are shown in Table A2.

Trends of yield of not-for-sale Chinese cabbage and cabbage from 2009 to 2018 are shown in Figure 1 and Table A2. In Nagano Prefecture, the not-for-sale yield of Chinese cabbage in 2012 was higher than in the preceding and following years. In Nagano Prefecture, it was 52.9 g/day/person in 2012 and between 31.6 and 44.5 g/day/person in other years (in 2016: 40.6 g/day/person). In the other prefectures, the range was between 10.7 and 13.1 g/day/person (in 2012: 10.8 g/day/person; in 2016: 12.4 g/day/person). In addition, in Gunma Prefecture, the not-for-sale yield of cabbage in 2012 was higher than in the other years. In Gunma Prefecture, it was 45.7 g/day/person in 2012, and ranged between 24.4 and 35.1 g/day/person in other years (in 2016: 24.4 g/day/person). In the other prefectures, the range was 5.8–6.8 g/day/person (in 2012: 6.3 g/day/person; in 2016: 5.8 g/day/person).

### 3.2. Parallel Trends

The results of the confirmation of parallel trends are shown in Table 2. The coefficients (95% CIs) of interaction of Chinese cabbage and Chinese cabbage with pickled leaves were 1.000 (−2.915–4.916) and 1.006 (−3.249–5.261), respectively. The coefficient (95% CI) of interaction of cabbage was −10.194 (−14.680 to −5.706). Interactions were small for Chinese cabbage and Chinese cabbage with pickled leaves as the dependent variable. However, the interaction with cabbage as the dependent variable was large. Therefore, only Chinese cabbage and Chinese cabbage with pickled leaves were used in the main analysis.

### 3.3. Main Analyses (Difference-in-Differences)

**Figure 2:**
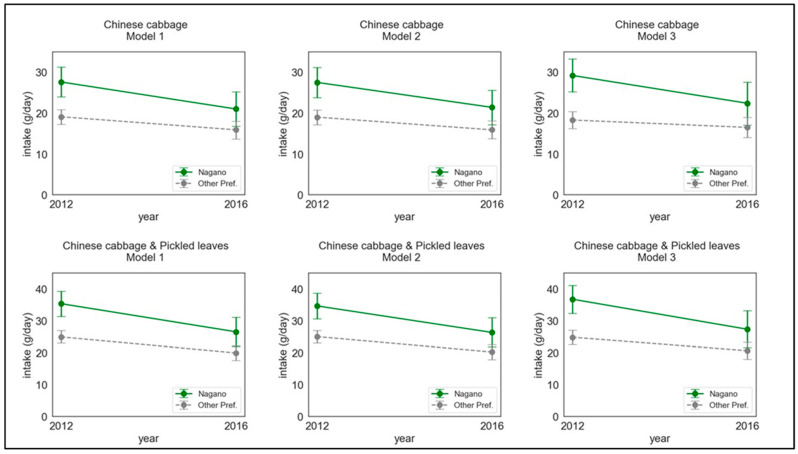
Trends of Chinese cabbage intake according to prefectures. Nagano: only Nagano Prefecture; Other pref.: prefectures among *Kanto* 2 other than Nagano (i.e., Ibaraki, Tochigi, Gunma, and Yamanashi). Details of the data are shown in Table A3.

The results of difference-in-differences analyses are shown in Table 3, Figure 2, and Table A3. For Chinese cabbage, interaction coefficients (95% CIs) of Models 1, 2, and 3 were −3.380 (−9.590–2.829), −2.991 (−9.217–3.236), and −5.032 (−12.406–2.342), respectively. For Chinese cabbage with pickled leaves, interaction coefficients (95% CIs) of Models 1, 2, and 3 were −3.380 (−9.590–2.829), −2.991 (−9.217–3.236), and −5.032 (−12.406–2.342), respectively. From 2012 to 2016, Chinese cabbage intake decreased in both Nagano and the other prefectures. However, the decrease was larger in Nagano in all models. These trends were similar when using the Chinese cabbage with pickled leaves as the dependent variable. For example, in Model 1 with Chinese cabbage as the dependent variable, Nagano decreased by 6.6 g (from 27.6 g in 2012 to 21.0 g in 2016), while the other prefectures decreased by 3.2 g (from 19.1 g in 2012 to 15.9 g in 2016).

## 4. Discussion

This study examined the impact of prefecture-level yield of not-for-sale vegetables on vegetable intake among a Japanese population. I regarded the increase in the yield of Chinese cabbage and cabbage that were not shipped in Nagano or Gunma Prefecture in 2012 as a natural experiment. The years 2012 and 2016 were the large-scale survey years of NHNS. Therefore, the effect of the change in prefecture-level yield of not-for-sale vegetables was evaluated by comparing the changes in intake in each prefecture between 2012 and 2016. Parallel trends were checked using a second control group, and no parallel trends were identified for cabbage. The results of the main analyses highlighted that the interaction between year and prefecture on the intake of Chinese cabbage was small. From 2012 to 2016, Chinese cabbage intake decreased in both Nagano and the other prefectures; however, the decrease was larger in Nagano. A number of reports have confirmed the relationship between the prefecture-level yield of not-for-sale vegetables and vegetable intake [23,24]. However, none have examined the causal relationship, and this is the first study to verify a causal relationship. Increased vegetable intake lowers the risk of certain chronic diseases and death [1,2,3,4,5,6,7,8]. The results of this study provide an important resource for improving health through vegetable intake in rural Japan.

The decrease in Chinese cabbage intake between 2012 and 2016 was approximately twice as large in Nagano compared to other prefectures. The absolute amount was approximately from 3 to 5 g/day/person. A previous study has estimated that a 1 g/day/person increase in the yield of prefecture-level not-for-sale vegetables increases prefectural residents’ vegetable intake by 0.390 g/day/person (95% CI: 0.183−0.596) [24]. The difference in the yield of not-for-sale Chinese cabbage between Nagano and other prefectures in this study was 42.1 g/day/person (52.9–10.8) in 2012, and 28.2 g/day/person (40.6–12.4) in 2016. Therefore, the difference decreased by 13.9 g/day/person from 2012 to 2016. Using the estimates from the previous study [24], it is estimated that a difference of 5.4 g/day/person (95% CI: 2.5–8.3) in the decrease in Chinese cabbage intake between 2012 and 2016 between Nagano and other prefectures would occur. In light of this value, it is valid that the decrease was approximately 3–5 g greater in Nagano Prefecture in this study.

A previous study has reported high vegetable intake in rural areas of Gunma Prefecture, Japan, despite poor access to stores selling food products, and has suggested that part of this is contributed by home consumption and received food [21]. When the results of this study and those of the previous study [24] are combined, the amount of yield of not-for-sale vegetables contributing to vegetable intake is minimal. Considering cost-effectiveness, it is inappropriate to adopt a policy of growing large quantities of not-for-sale vegetables. However, modestly maintaining the small contribution from the yield of not-for-sale vegetables that currently exists is important for maintaining a healthy dietary intake for rural residents. However, the number of farmers and cultivated land areas are reducing in Japan [34,35]. The agricultural support policies that would break out of this situation are necessary to maintain a modest distribution of not-for-sale crops in Japanese rural areas.

### Limitations

This study has several limitations. First, similar to previous studies, these results only pertain to Japan [23,24]. In Japan, it is common to share one’s produce surplus with neighbors. Therefore, the results of similar studies will differ in countries and regions where there is no such accepted culture. Consequently, in this study, only the yield of not-for-sale vegetables produced by farmers was used. The crops produced by nonfarmers in home gardens and community gardens were excluded. In fact, many studies have shown that vegetable intake increases due to home gardening and community gardening activities [9,10,11,12,13,14,15,16,17]. Therefore, future studies should use a method to estimate the amount of not-for-sale vegetable yield by also including these. In addition, this study examined data at the prefecture-level. However, the transfer of surplus often appears within smaller regional units or social connections among people not in the local community. Therefore, further micro-scale regional or social community-based studies are required. Finally, this study focused on Chinese cabbage, for which a natural experimental environment was accidentally identified. (Cabbage was excluded in the main analysis because no parallel trend was identified, but crude values calculated from the NHNS data were provided as a supplement file (Table S1).) Therefore, future studies should analyze other vegetables and fruits to discover similar trends.

## 5. Conclusions

This study examined the impact of the prefecture-level yield of not-for-sale vegetables on vegetable intake in Japan, focusing on Chinese cabbage intake. I confirmed whether the significant increase in the harvest of not-for-sale Chinese cabbage in Nagano Prefecture in 2012 affected the amount of Chinese cabbage intake among Nagano residents by comparing it to 2016. Consequently, the interaction between year and prefecture was minor. From 2012 to 2016, Chinese cabbage intake decreased in both Nagano and the other prefectures; however, the decrease was larger in Nagano. In conclusion, the effect of the prefecture-level yield of not-for-sale vegetables on vegetable intake might be small.

## Figures and Tables

**Table 1:** Information of subjects among *Kanto* 2.

	*Survey Year*			
	2012		2016	
	n	%	n	%
	3369		2425	
*Prefecture*				
Ibaraki	795	23.6	422	17.4
Tochigi	713	21.2	708	29.2
Gunma	669	19.9	439	18.1
Yamanashi	538	16.0	352	14.5
Nagano	654	19.4	504	20.8
*Gender*				
Men	1606	47.7	1152	47.5
Women	1763	52.3	1273	52.5
*Age*				
20–39	749	22.2	510	21.0
40–59	1217	36.1	861	35.5
60–79	1403	41.6	1054	43.5
*Living style*				
Living alone	215	6.4	239	9.9
Living together	3154	93.6	2186	90.1
*Employment*				
Not agricultural worker	3103	92.1	2284	94.2
Agricultural worker	266	7.9	135	5.6
(Missing)	0	0.0	6	0.2
*Energy intake*				
1st quartile group	860	25.5	595	24.5
2nd quartile group	781	23.2	623	25.7
3rd quartile group	837	24.8	601	24.8
4th quartile group	891	26.4	606	25.0
*Body mass index*				
<18.5	179	5.3	136	5.6
18.5 to <25.0	1801	53.5	1176	48.5
≥25.0	711	21.1	531	21.9
(Missing)	678	20.1	582	24.0
*Alcohol drinking*				
Rarely or never	1718	51.0	1309	54.0
4 days/week or less	839	24.9	523	21.6
5 days/week or more	765	22.7	553	22.8
(missing)	47	1.4	40	1.6

**Table 2:** Confirmation of parallel trends.

	Chinese Cabbage (n = 8043)		Chinese Cabbage and Pickled Leaves (n = 8043)		Cabbage (n = 8093)	
	Coef.	95% CI	Coef.	95% CI	Coef.	95% CI
Interaction (year × area)	1.000	(−2.915, 4.916)	1.006	(−3.249, 5.261)	−10.194	(−14.680, −5.706)
Year	2.228	(−0.729, 5.185)	4.047	(0.833, 7.260)	3.039	(−0.361, 6.439)
Area	1.769	(−1.151, 4.689)	3.434	(0.260, 6.607)	5.953	(2.620, 9.286)
(Intercept)	14.095	(11.934, 16.255)	16.436	(14.087, 18.783)	29.745	(27.259, 32.229)

General linear models. Year: 2012 (ref.) and 2016; area: *Kanto* 2 (ref.) and *Kanto* 1. Coef.: regression coefficients; CI: confidence intervals.

**Table 3:** Interaction of year and prefecture on Chinese cabbage intake.

	Model 1		Model 2		Model 3	
	Coef.	95% CI	Coef.	95% CI	Coef.	95% CI
*Chinese cabbage*						
Interaction (year × prefecture)	−3.380	(−9.590, 2.829)	−2.991	(−9.217, 3.236)	−5.032	(−12.406, 2.342)
Year	6.609	(1.060, 12.156)	6.075	(0.517, 11.632)	6.862	(0.243, 13.480)
Prefecture	−5.138	(−9.822, −0.453)	−5.465	(−10.20, −0.728)	−5.877	(−11.726, −0.026)
(Intercept)	21.002	(16.832, 25.170)	16.052	(8.199, 23.903)	15.703	(4.227, 27.179)
*Chinese cabbage and Pickled leaves*						
Interaction (year × prefecture)	−3.684	(−10.420, 3.052)	−3.495	(−10.247, 3.256)	−5.218	(−13.271, 2.835)
Year	8.736	(2.718, 14.754)	8.286	(2.260, 14.312)	9.355	(2.127, 16.582)
Prefecture	−6.658	(−11.739, −1.576)	−6.143	(−11.278, −1.006)	−6.726	(−13.114, −0.338)
(Intercept)	26.528	(22.004, 31.050)	22.171	(13.656, 30.685)	18.291	(5.7584, 30.822)

General liner models. Year: 2012 (ref.) and 2016; prefecture: Nagano and other prefectures (ref.). Coef.: regression coefficients; CI: confidence intervals. Model 1: Crude models (n = 5794). Model 2: Adjusted for gender, age, living style, employment, and energy intake (n = 5788). Model 3: Adjusted for body mass index, and alcohol drinking added to Model 2 (n = 4502).

## Data Availability

The raw data are not publicly available due to ethical restrictions.

## Supplementary Materials

The following supporting information can be downloaded at: https://www.mdpi.com/article/10.3390/nu14142884/s1, Table S1: Cabbage intake according to prefectures.

## Appendix A

**Table A1:** Information of subjects among *Kanto* 1.

	*Survey Year*			
	2012		2016	
	N	%	n	%
	1819		1588	
*Prefecture*				
Saitama	572	31.4	515	32.4
Chiba	481	26.4	532	33.5
Tokyo	366	20.1	300	18.9
Kanagawa	400	22.0	241	15.2
*Gender*				
Men	850	46.7	740	46.6
Women	969	53.3	848	53.4
*Age*				
20–39	479	26.3	339	21.3
40–59	637	35.0	567	35.7
60–79	703	38.6	682	42.9
*Living style*				
Living alone	173	9.5	204	12.8
Living together	1646	90.5	1384	87.2
*Employment*				
Not agricultural worker	1794	98.6	1553	97.8
Agricultural worker	25	1.4	31	2.0
(Missing)	0	0.0	4	0.3
*Energy intake*				
1st quartile group	435	23.9	411	25.9
2nd quartile group	486	26.7	410	25.8
3rd quartile group	450	24.7	412	25.9
4th quartile group	448	24.6	355	22.4
*Body mass index*				
<18.5	128	7.0	109	6.9
18.5 to <25.0	1101	60.5	861	54.2
≥25.0	382	21.0	307	19.3
(Missing)	208	11.4	311	19.6
*Alcohol drinking*				
Rarely or never	854	46.9	784	49.4
4 day/week or less	534	29.4	436	27.5
5 day/week or more	408	22.4	337	21.2
(Missing)	23	1.3	31	2.0

**Table A2:** Trends of yield of not-for-sale Chinese cabbage and cabbage.

	Chinese Cabbage		Cabbage	
Year	Nagano	Other Pref.	Gunma	Other Pref.
2009	44.5	10.9	34.0	6.8
2010	34.3	10.7	32.6	6.4
2011	38.4	11.1	31.2	6.8
2012	52.9	10.8	45.7	6.3
2013	33.2	10.9	33.9	6.2
2014	33.2	11.7	33.5	6.1
2015	33.0	11.5	28.5	6.1
2016	40.6	12.4	24.4	5.8
2017	33.7	12.8	34.0	6.8
2018	31.6	13.1	35.1	6.6

Not-for-sale yield (g/day/person). Nagano: only Nagano Prefecture; Gunma: only Gunma Prefecture; Other pref.: for Chinese cabbage, mean of prefectures among *Kanto* 2 other than Nagano (i.e., Ibaraki, Tochigi, Gunma, and Yamanashi). For cabbage, mean of prefectures among *Kanto* 2 other than Gunma (i.e., Ibaraki, Tochigi, Yamanashi, and Nagano).

**Table A3:** Trends of Chinese cabbage intake according to prefectures.

		Chinese Cabbage		Chinese Cabbage and Pickled Leaves	
Year	Prefecture	M	95% CI	M	95% CI
*Model 1*					
2012	Nagano	27.6	(23.9, 31.2)	35.3	(31.2, 39.2)
	Other Pref.	19.1	(17.2, 20.8)	24.9	(22.9, 26.8)
2016	Nagano	21.0	(16.8, 25.1)	26.5	(22.0, 31.0)
	Other Pref.	15.9	(13.7, 17.9)	19.9	(17.5, 22.1)
*Model 2*					
2012	Nagano	27.5	(23.8, 31.1)	34.6	(30.6, 38.5)
	Other Pref.	19.0	(17.2, 20.8)	25.0	(23.0, 26.9)
2016	Nagano	21.4	(17.2, 25.6)	26.3	(21.7, 30.8)
	Other Pref.	15.9	(13.7, 18.0)	20.2	(17.8, 22.4)
*Model 3*					
2012	Nagano	29.2	(25.1, 33.2)	36.7	(32.2, 41.1)
	Other Pref.	18.3	(16.2, 20.3)	24.8	(22.4, 27.0)
2016	Nagano	22.4	(17.0, 27.6)	27.3	(21.5, 33.0)
	Other Pref.	16.5	(13.9, 18.9)	20.6	(17.9, 23.3)

M: Estimated mean; CI: confidence intervals. Nagano: only Nagano Prefecture; Other pref.: prefectures among *Kanto* 2 other than Nagano (i.e., Ibaraki, Tochigi, Gunma, and Yamanashi). Model 1: Crude models (n = 5794). Model 2: Adjusted for gender, age, living style, employment, and energy intake (n = 5788). Model 3: Adjusted for body mass index, and alcohol drinking added to Model 2 (n = 4502).

## References

[1] Ma L., Sun Z., Zeng Y., Luo M., Yang J. (2018). Molecular mechanism and health role of functional ingredients in blueberry for chronic disease in human beings. Int. J. Mol. Sci..

[2] Zeng Y., Li Y., Yang J., Pu X., Du J., Yang X., Yang T., Yang S. (2017). Therapeutic role of functional components in alliums for preventive chronic disease in human being. Evid. Based Complement. Altern. Med..

[3] Aune D., Giovannucci E., Boffetta P., Fadnes L.T., Keum N., Norat T., Greenwood D.C., Riboli E., Vatten L.J., Tonstad S. (2017). Fruit and vegetable intake and the risk of cardiovascular disease, total cancer and all-cause mortality-a systematic review and dose-response meta-analysis of prospective studies. Int. J. Epidemiol..

[4] He F.J., Nowsonk C.A., Lucas M., MacGregor G.A. (2007). Increased consumption of fruit and vegetables is related to a reduced risk of coronary heart disease: Meta-analysis of cohort studies. J. Hum. Hypertens..

[5] He F.J., Nowsonk C.A., Lucas M., MacGregor G.A. (2006). Fruit and vegetable consumption and stroke: Meta-analysis of cohort studies. Lancet..

[6] Guzek D., Głąbska D., Groele B., Gutkowska K. (2021). Fruit and vegetable dietary patterns and mental health in women: A systematic review. Nutr. Rev..

[7] Ju S.Y., Park Y.K. (2019). Low fruit and vegetable intake is associated with depression among Korean adults in data from the 2014 Korea National Health and Nutrition Examination Survey. J. Health Popul. Nutr..

[8] World Health Organization Healthy Diet. https://www.who.int/news-room/fact-sheets/detail/healthy-diet.

[9] Garcia M.T., Ribeiro S.M., Germani A.C.C.G., Bógus C.M. (2018). The impact of urban gardens on adequate and healthy food: A systematic review. Public Health Nutr..

[10] Schram-Bijkerk D., Otte P., Dirven L., Breure A.M. (2018). Indicators to support healthy urban gardening in urban management. Sci. Total Environ..

[11] Langellotto G., Gupta A. (2012). Gardening increases vegetable consumption in school-aged children: A meta-analytical synthesis. HortTechnology.

[12] McCormack L.A., Laska M.N., Larson N.I., Story M. (2010). Review of the nutritional implications of farmers’ markets and community gardens: A call for evaluation and research efforts. J. Am. Diet. Assoc..

[13] Robinson-O’Brien R., Story M., Heim S. (2009). Impact of garden-based youth nutrition intervention programs: A review. J. Am. Diet. Assoc..

[14] Machida D., Kushida O. (2020). The influence of food production experience on dietary knowledge, awareness, behaviors, and health among Japanese: A systematic review. Int. J. Environ. Res. Public Health.

[15] Savoie-Roskos M.R., Wengreen H., Durward C. (2017). Increasing fruit and vegetable intake among children and youth through gardening-based interventions: A systematic review. J. Acad. Nutr. Diet..

[16] Ohly H., Gentry S., Wigglesworth R., Bethel A., Lovell R., Garside R. (2016). A systematic review of the health and well-being impacts of school gardening: Synthesis of quantitative and qualitative evidence. BMC Public Health.

[17] Davis J.N., Spaniol M.R., Somerset S. (2015). Sustenance and sustainability: Maximizing the impact of school gardens on health outcomes. Public Health Nutr..

[18] Tatebayashi K., Kamiyama C., Matsui T., Saito O., Machimura T. (2019). Accounting shadow benefits of non-market food through food-sharing networks on Hachijo Island, Japan. Sustain. Sci..

[19] Saito O., Havas J., Shirai K., Kurisu K., Aramaki T., Hanaki K. (2015). Non-Market food provisioning services in Hachijo Island, Japan and their implications toward building a resilient island. J. Jpn. Soc. Civ. Eng. Ser. G.

[20] Umezawa A., Miwa T., Shibui E., Namikawa T., Tanaka N., Ishikawa M. (2012). Total vegetable intake and homegrown vegetable intake in the rural area residents of Hokkaido. Eiyogaku Zassi.

[21] Machida D., Yoshida T. (2018). Vegetable intake frequency is higher among the rural than among the urban or suburban residents, and is partially related to vegetable cultivation, receiving, and purchasing at farmers’ markets: A cross-sectional study in a city within Gunma, Japan. J. Rural. Med..

[22] Machida D., Yoshida T. (2018). Negative association of vegetable cultivation with the proportion of severely insufficient vegetable intake both directly and indirectly: A cross-sectional study in a city in Gunma, Japan. J. Rural. Med..

[23] Machida D., Kushida O., Yoshida T. (2017). Relationship between vegetable intake and vegetable cultivation by prefecture in Japan: An ecological study at the prefectural level. Nihon Kenkoukyouiku Gakkaishi.

[24] Machida D. (2021). Relationship between Prefecture-Level Yield of Not-for-Sale Fruits and Vegetables and Individual-Level Fruit and Vegetable Intake in Japan: A Cross-Sectional Study. Nutrients.

[25] Ministry of Agriculture, Forestry and Fisheries Outline of National Crop Survey (Vegetables). https://www.maff.go.jp/j/tokei/kouhyou/sakumotu/sakkyou_yasai/gaiyou/index.html#1.

[26] Ministry of Health, Labor and Welfare (2016). The National Health and Nutrition Survey in Japan. https://www.mhlw.go.jp/content/000681180.pdf.

[27] Ministry of Health, Labor and Welfare (2012). The National Health and Nutrition Survey in Japan. https://www.mhlw.go.jp/bunya/kenkou/eiyou/dl/h24-houkoku.pdf.

[28] Brittin J., Frerichs L., Sirard J.R., Wells N.M., Myers B.M., Garcia J., Sorensen D., Trowbridge M.J., Huang T. (2017). Impacts of active school design on school-time sedentary behavior and physical activity: A pilot natural experiment. PLoS ONE.

[29] Petimar J., Moran A.J., Ramirez M., Block J.P. (2020). A Natural Experiment to Evaluate the Nutritional Content of Restaurant Meal Purchases After Calorie Labeling. J. Acad. Nutr. Diet..

[30] Barlow P., McKee M., Basu S., Stuckler D. (2017). Impact of the North American Free Trade Agreement on high-fructose corn syrup supply in Canada: A natural experiment using synthetic control methods. CMAJ.

[31] Agriculture & Livestock Industries Corporation Handouts for the 2nd Vegetable Supply and Demand Council Meeting in FY2012 (Document 1–2: Implementation of Emergency Supply and Demand Adjustment Project for Cabbage and Chinese Cabbage). https://www.alic.go.jp/content/000088408.pdf.

[32] Statistics Bureau Ministry of Internal Affairs and Communications (2015). Summary of Census Results. https://www.stat.go.jp/data/kokusei/2015/kekka/kihon1/pdf/gaiyou1.pdf.

[33] Suzuki W., Iwamoto Y., Yuda M., Morozumi R. (2015). Measuring the effect of specific health checkups and specific maintenance guidance in Japan: Evidence from program evaluation approaches. Jpn. J. Health Econ. Policy.

[34] Japanese Ministry of Agriculture, Forestry and Fisheries Statistics on the Agricultural Labor Force. http://www.maff.go.jp/j/tokei/sihyo/data/08.html.

[35] Japanese Ministry of Agriculture, Forestry and Fisheries Area Survey. http://www.maff.go.jp/j/tokei/kouhyou/sakumotu/menseki/index.html#r.

