# Supplementary material for: Influence of Prefecture-Level Yield of Not-for-Sale Vegetables on Vegetable Intake in Japan: A Natural Experiment
Source: Nutrients. 2022 Jul 14;14(14):2884. doi: 10.3390/nu14142884 (PMC9323108; doi:10.3390/nu14142884)
Supplement: Supplementary file 1 [file nutrients-14-02884-s001.zip › nutrients-1808596-supplementary.pdf]

Supplement file

Influence of Prefecture-Level Yield of Not-for-Sale Vegetables on Vegetable Intake in Japan: A Natural Experiment

Daisuke Machida

Table S1. Cabbage intake according to prefectures.

year	Prefecture	M	95% CI
2012	Gunma	27.1	(23.6, 30.4)
	Other Pref.	28.5	(26.7, 30.3)
2016	Gunma	32.4	(27.7, 37.1)
	Other Pref.	35.7	(33.3, 38.0)

N = 5794; M: Estimated means; CI: confidence intervals
Gunma: only Gunma Prefecture; Other Pref.: mean of prefectures among *Kanto* 2 other than Nagano (i.e., Ibaraki, Tochigi, Nagano, and Yamanashi).
Subjects were men and women aged 20–79 years living in the Kanto 2 area. Those who did not respond to food intake status, and pregnant and lactating women, whose diets may have been significantly affected, were excluded from the analysis. Subjects with energy intakes below 800 kcal and over 3000 kcal (approximately mean ± 2 SD) were also excluded.

Note. Gunma increased by 5.3g (from 27.1g in 2012 to 32.4g in 2016), while the other prefectures increased by 7.2g (from 28.5g in 2012 to 35.7g in 2016). Because the increase was slightly smaller in Gunma than in the other prefectures, it could be interpreted that there was a slight influence of the increased yield non-for-sale in 2012. However, since the parallel trend remains unconfirmed as a premise, it is not mentioned in the text.
